# Efficacy of Neoadjuvant Targeted Therapy for Borderline Resectable III B-D or IV Stage BRAF ^V600^ Mutation-Positive Melanoma

**DOI:** 10.3390/cancers14010110

**Published:** 2021-12-27

**Authors:** Anna M. Czarnecka, Krzysztof Ostaszewski, Aneta Borkowska, Anna Szumera-Ciećkiewicz, Katarzyna Kozak, Tomasz Świtaj, Paweł Rogala, Iwona Kalinowska, Hanna Koseła-Paterczyk, Konrad Zaborowski, Paweł Teterycz, Andrzej Tysarowski, Donata Makuła, Piotr Rutkowski

**Affiliations:** 1 Department of Soft Tissue/Bone Sarcoma and Melanoma, Maria Sklodowska-Curie National Research Institute of Oncology, 02-781 Warsaw, Poland; Krzysztof.Ostaszewski@pib-nio.pl (K.O.); aneta.borkowska@pib-nio.pl (A.B.); katarzyna.kozak@pib-nio.pl (K.K.); tswitaj@coi.pl (T.Ś.); pawel.rogala@pib-nio.pl (P.R.); iwona.kalinowska@pib-nio.pl (I.K.); hanna.kosela-paterczyk@pib-nio.pl (H.K.-P.); konrad.zaborowski@pib-nio.pl (K.Z.); pawel.teterycz@pib-nio.pl (P.T.); piotr.rutkowski@pib-nio.pl (P.R.); 2 Department of Pathology and Laboratory Medicine, Maria Sklodowska-Curie National Research Institute of Oncology, 02-781 Warsaw, Poland; anna.szumera-cieckiewicz@pib-nio.pl (A.S.-C.); andrzej.tysarowski@pib-nio.pl (A.T.); 3 Department of Molecular and Translational Oncology, Maria Sklodowska-Curie National Research Institute of Oncology, 02-781 Warsaw, Poland; 4 Department of Radiology I, Maria Sklodowska-Curie National Research Institute of Oncology, 02-781 Warsaw, Poland; donata.makula@pib-nio.pl

**Keywords:** melanoma, neoadjuvant, BRAF, targeted therapy

## Abstract

**Simple Summary:**

Neoadjuvant therapy for locally advanced disease or potentially resectable metastatic melanoma is expected to improve operability and clinical outcomes over upfront surgery. 46 patients were treated with BRAFi/MEKi or BRAFi before surgery with 78% R0 resection. In patients with a major pathological response with no, or less than 10%, viable cells in the tumor, median DFS and PFS were significantly longer than in patients with a minor pathological response.

**Abstract:**

Neoadjuvant therapy for locally advanced disease or potentially resectable metastatic melanoma is expected to improve operability and clinical outcomes over upfront surgery and adjuvant treatment as it is for sarcoma, breast, rectal, esophageal, or gastric cancers. Patients with locoregional recurrence after initial surgery and those with advanced regional lymphatic metastases are at a high risk of relapse and melanoma-related death. There is an unmet clinical need to improve the outcomes for such patients. Patients with resectable bulky stage III or resectable stage IV histologically confirmed melanoma were enrolled and received standard-dose BRAFi/MEKi for at least 12 weeks before feasible resection of the pre-therapy target and then received at least for the next 40 weeks further BRAFi/MEKi. Of these patients, 37 were treated with dabrafenib and trametinib, three were treated with vemurafenib and cobimetinib, five with vemurafenib, and one with dabrafenib alone. All patients underwent surgery with 78% microscopically margin-negative resection (R0) resection. Ten patients achieved a complete pathological response. In patients with a major pathological response with no, or less than 10%, viable cells in the tumor, median disease free survival and progression free survival were significantly longer than in patients with a minor pathological response. No patient discontinued neoadjuvant BRAFi/MEKi due to toxicity. BRAFi/MEKi pre-treatment did not result in any new specific complications of surgery. Fourteen patients experienced disease recurrence or progression during post-operative treatment. We confirmed that BRAFi/MEKi combination is an effective and safe regimen in the perioperative treatment of melanoma. Pathological response to neoadjuvant treatment may be considered as a surrogate biomarker of disease recurrence.

## 1. Introduction

Neoadjuvant therapy is currently the standard of care in some locally advanced solid tumors, including sarcomas, breast, rectal, esophageal, and gastric cancers [1,2,3,4,5,6]. In the melanoma field, the neoadjuvant approach is still a matter of debate and has not been included in treatment guidelines as recommended treatment with proof level IA [7,8]. Currently, standard management of locoregional and oligometastatic melanoma is surgery followed by systemic adjuvant therapy. Similarly, complete excision is the therapy of choice for isolated and resectable local and regional recurrence [7,9]. About five to 15% of stage III melanoma patients are unable to undergo up-front resection due to the extent of their tumors, location of the tumor, and/or the anticipated morbidity of the surgery. In such cases, standard care with surgical resection followed by adjuvant treatment is impossible [10,11]. Moreover, up to 10% of melanoma patients develop locoregional recurrence [12]. In real-world practice, patients may present with advanced and/or unresectable disease locally at the primary tumor site, but also in the regional nodal basin or basis, as well as within the dermal lymphatic channels between the primary melanoma and regional lymph nodes. Such patients pose a significant challenge for the surgeon [13]. Technically unresectable melanoma refers to cases for whom surgical resection is not possible without unacceptable functional impairment; as well as cases macroscopically resectable, but of high-volume or multifocal disease, which bears a high likelihood of residual micro-or macroscopic disease after surgery; or finally, those with encasement of vital structures and cases in which increased risk of major adverse events during surgery is anticipated [11,14]. Patients with borderline resectable melanoma are those with a primary or recurrent disease with advanced regional infiltration that makes surgical resection challenging [15]. In general, resection of more advanced disease is associated with increased perioperative morbidity and may lead to incomplete surgical resection. Moreover, it was described that a significant number of patients with advanced regional melanoma harbor radiographically and clinically occult regional micro-metastases and/or systemic disease and, therefore, subsequently quickly relapse not only locally but also with distant metastases [16,17]. For all these groups of patients, neoadjuvant systemic therapy may be a reasonable treatment strategy.

In general, among patients with stage III locoregional metastases, only 77% are expected to be alive at five years [18]. For melanoma stage III A-D, as per eight editions of the AJCC staging system, between 93% and 32% of patients are alive at five years. In fact, patients with palpable regional lymphatic metastases/stage IIIC-D are at the highest high risk of relapse and melanoma-related death exceeding 70% at five years [19,20]. In the European/EORTC cohort for stage IIIA, the melanoma-specific survival (MSS) is even lower, with MSS rate at five years of 80%, and at ten years 71%; for stage IIIB, these figures are 75% and 61% [21]. Patients who develop regional recurrence after initial surgery have a nearly 50% mortality rate at five years after such local recurrence, and only about 30% are alive at ten years [22]. With the use of targeted adjuvant therapy, relapse-free survival for stage III patients is longer but still unsatisfactory. The median recurrence-free survival (RFS) rate is about 80% at the first year, almost 60% after three years, and 52% at five years [23,24]. Such treatment demonstrated higher 3-years OS-rates than placebo (86% vs. 77% HR 0.57; 95%CI= 0.42–0.79) [25]. In fact, clinical benefit from dabrafenib/trametinib is consistent regardless of lymph node (LN) involvement or melanoma ulceration, apart from stage IIIA cases where the upper confidence interval is marginally crossed (HR 0.58; 95% CI= 0.32–1.06). Moreover, adjuvant therapy in non-ulcerated melanomas with macro-metastases is associated with the smallest RFS benefit and does not reach statistical significance (HR 0.73; 95%CI= 0.50–1.05) [25]. Moreover, after adjuvant therapy for stage III melanoma, the risk of relapse still remains significant, mostly in patients with initially palpable or radiographically detected nodal metastases [26,27]. Therefore there is still an unmet need to improve the outcomes of stage III melanoma patients, as well as those with local recurrence and borderline resectable cases. Due to the high response rate, rapid response kinetics, and favorable drug safety profile in metastatic and adjuvant settings, targeted therapy with BRAF and MEK inhibitors may also provide an effective neoadjuvant treatment in melanoma patients [28,29].

From a clinical point of view, neoadjuvant treatment accompanied by pathological evaluation potentially enables to selection of patients that are the most likely to respond to treatment, such as those with more favorable melanoma tumor biology [13]. Based on the experience of other malignancies, neoadjuvant therapy for locally advanced and potentially resectable metastatic melanoma is expected to improve long-term outcomes over upfront surgery and adjuvant treatment [26,27]. For neoadjuvant therapy, it is expected that a complete clinical response to treatment may be achieved with a low risk of losing regional control [28]. For all the reasons described above, there is an increasing interest in the role of neoadjuvant targeted therapies for melanoma patients. At this moment, 48 active, planned, or ongoing trials on neoadjuvant therapies in high-risk melanoma are reported (Clinicaltrials.gov, 19 September 2021). As reported in patients with *BRAF V600E* or *V600K* mutant stage III melanoma treated with 12-months adjuvant dabrafenib + trametinib, RFS is 52% at 5-years of follow-up [24]. Released data from the NeoCombi (NCT01972347) phase 2 trial suggest that neoadjuvant targeted therapy with BRAF and MEK inhibitors (BRAFi/MEKi) is feasible and improves patients outcomes. The reported toxicity of neoadjuvant treatment with BRAFi/MEKi was acceptable and similar to that seen in patients treated for advanced disease. For dabrafenib and trametinib therapy, the major toxicities reported were fevers, chills, and headache; with grade 3–4 adverse events (AEs) in 29% of patients in the Neo Combi trial [30] and 15% of patients of grade 3 AEs in the REDUCTOR trial [10]. It is widely accepted that more research and reports of ongoing trials are needed to describe the efficacy of neoadjuvant melanoma treatment and to develop the new standard of care for patients with locally advanced melanoma [31]. In our study, we aim to describe the efficacy of neoadjuvant BRAF-oriented targeted therapy in patients with borderline resectable melanoma in real-world clinical practice.

## 2. Materials and Methods

### 2.1. Patients and Clinical Data

Patients with borderline resectable bulky stage III or resectable stage IV histologically confirmed melanoma were enrolled in the study (Table 1). All cases were revised by a multidisciplinary team (MDT). *BRAF V600* mutation status and pathological diagnosis were confirmed with a validated genetic test by experienced molecular biology specialists and melanoma pathologists in one melanoma reference center. Eligible patients were ECOG PS  ≤ 1, and CT and/or PET-CT scans were performed at baseline. CT/PET-CT monitoring was continued 12 weekly thereafter. The pathological response (pR) was assessed in post-surgery specimens. The near-complete/major pR was defined as >0% but ≤10% viable melanoma cells, and minor pR included >10% viable melanoma cells as previously described [32]. Patients received standard-dose BRAFi/MEKi for at least 12 weeks prior to feasible resection of the pre-therapy target and then received for at least the next 40 weeks further BRAFi/MEKi. Patients were followed for at least 12 months, and the data cut-off was 30/October/2021. All deaths were assessed in the Polish Death Registry and National Cancer Registry.

### 2.2. Statistical Analysis

Patient and melanoma baseline characteristics were summarized using standard descriptive statistics: median (range) for continuous variables and frequency (proportion) for categorical variables. Progression free survival (PFS) was defined as the time from neoadjuvant therapy start to disease progression (preoperatively) or recurrence (postoperatively) or death from any cause. Disease free survival (DFS) was defined as the time from surgery to disease progression (preoperatively) or recurrence (postoperatively) or death from any cause. Overall survival (OS) was defined as the time from neoadjuvant treatment start to death. Survival (PFS, DFS and OS) was analyzed using the Kaplan-Meier method. Association between variables was tested using chi-squared or Fisher’s exact tests as appropriate. All statistical analyses were performed using R version 3.6.1 (R Core Team, www.r-project.org (accessed on 24 November 2021)).

## 3. Results

### 3.1. Neoadjuvant Treatment and Surgery

**Figure 1:**
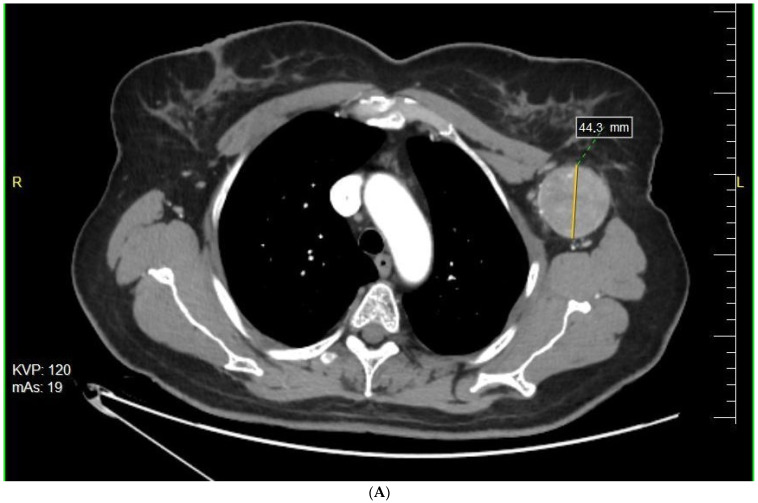

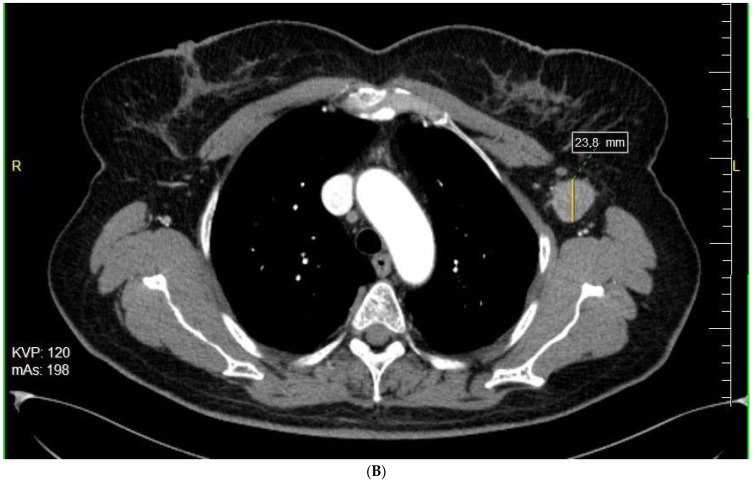
Response to preoperative BRAFi/MEKi therapy—the extent of the metastatic tumor in the left axilla before (**A**) and after (**B**) targeted therapy (Figure by Pawel Rogala).

Forty-six subsequent patients started neoadjuvant therapy with BRAF +/− MEK inhibitors between 1 October 2014 and 30 June 2020. At the time of neoadjuvant treatment initiation, 14 patients had disease recurrence, while 14 had extensive metastases in the regional lymph nodes (Table 2, Figure 1).

37 patients were treated with dabrafenib and trametinib, 3 patients were treated with vemurafenib and cobimetinib, 5 with vemurafenib monotherapy, and 1 with dabrafenib monotherapy. The median time of BRAFi/MEKi treatment before surgery was 16 weeks. All patients underwent surgery with 78% microscopically margin-negative resection (R0) resections. The therapeutic lymph node dissection (TLND) was performed in 18 patients. Other surgery procedures included metastasectomy and recurrence resection (Table 3).

**Figure 2:**
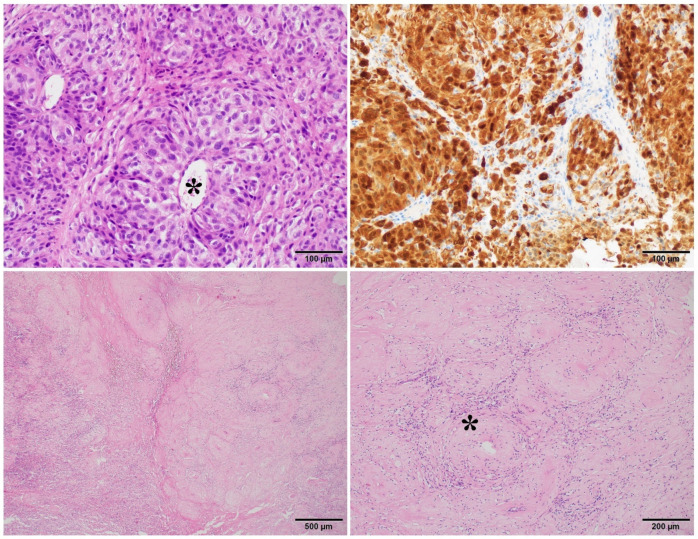
Response on BRAFi/MEKi therapy—the major pR: nearly no melanoma cells after treatment (bottom row) with a maintained histological pattern of melanoma growth around vessels (asterisk) highlighted by S100 immunohistochemical staining (upper row).

**Figure 3:**
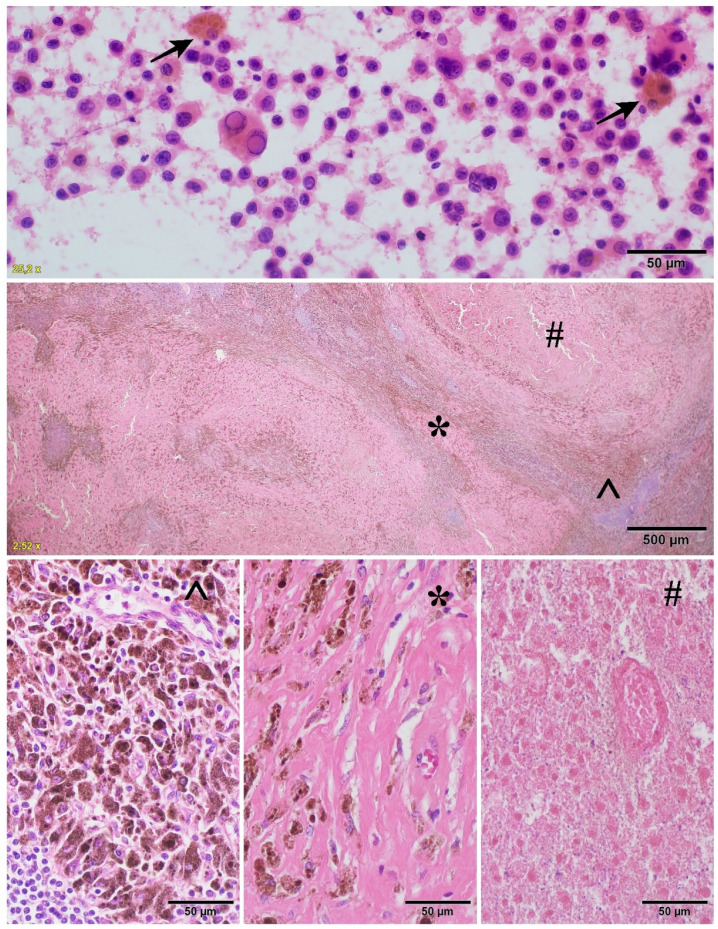
The major pR: a cytological image of melanoma (upper row) before treatment with numerous melano-phages (arrow) and after treatment (middle and bottom row) with melano-phages (^), fibrosis (*), and necrosis (#).

**Figure 4:**
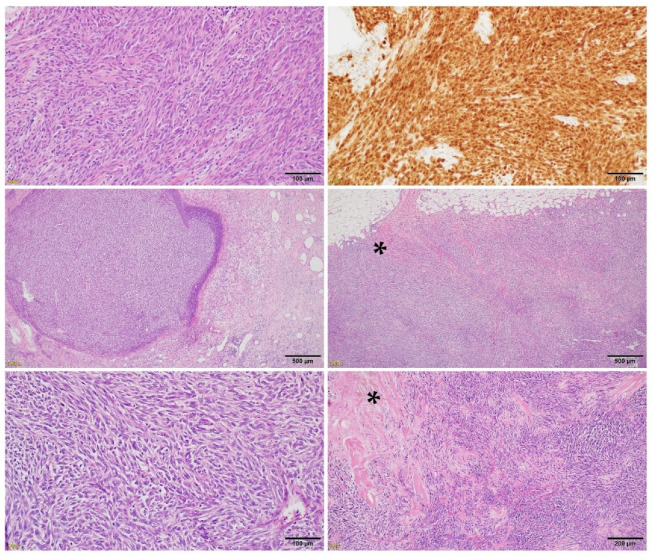
The minor pR: biopsy material of melanoma (upper row) before treatment with high S100 immunohistochemical expression and after treatment (middle and bottom row) with preserved melanoma infiltration and only focal fibrosis (*).

**Figure 5:**
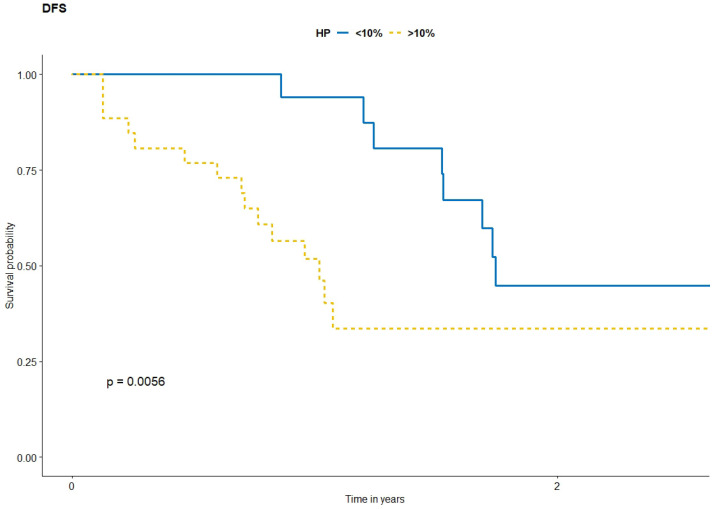
DFS after neoadjuvant treatment.

Ten patients achieved a complete pathological response; the subsequent ten patients had a major pathological response with less than 10% viable cells, while 26 had more viable melanoma cells in the post-treatment specimens, including one patient who did not respond to the treatment (all viable cells). In the major pR the viable melanoma cells were significantly diminished; necrosis with abundant fibrosis was predominantly detected (Figure 2 and Figure 3). In cases with minor pR, focal fibrosis was seen (Figure 4). In the whole group mDFS was 1.53 year and mPFS was 2.06 year, while mOS was not reached (Figure 5). Median time from diagnosis till last observation or death was 3.2 years. In patients with a pathological response of less than 10%, viable cells in the tumor observed median PFS and DFS were significantly longer than in patients with a higher number of viable melanoma cells in resected tumors, with HR = 1.68 (*p* = 0.019) and HR = 1.85 (*p* = 0.0056), respectively (Table 4). Median overall survival (OS) was not reached (HR = 1.77; *p* = 0.07). Neither PSF, DFS nor OS was dependent on age, sex, LDH activity, or neutrophil/lymphocyte ratio (NLR) at the time of treatment initiation (Table 1). In the whole group 91.1% (95%CI 0.832–0.998) was alive after 12 months, 75.6% (95%CI 0.633–0.903) after 2 years and 67.2% (95%CI 0.502–0.899) after 3 years.

Five patients were treated with radiation therapy due to R1 resection, including two with regional LND, one with regional recurrence after LND/SNLB, and two after primary tumor recurrence resection.

### 3.2. Neoadjuvant Treatment Safety

No patient discontinued BRAFi/MEKi due to toxicity, consent withdrawal, or progression during the neoadjuvant treatment period. BRAFi/MEKi pre-treatment did not result in any new specific complications of surgery. Early and late surgical complication frequencies were consistent with those reported in our patients treated with up-front surgery at the same stage of melanoma. The incidence of complications in the perioperative period was typical for patients undergoing lymphadenectomy (17%): wound dehiscence (five patients), wound suppuration (three patients). No treatment-related deaths were reported.

### 3.3. Treatment after Surgery

The median time on targeted therapy was 60 weeks, including a week-long perioperative off therapy period. At the analysis time, two patients experienced local recurrence after post-neoadjuvant surgery—both cases were patients with R1 resections (both treated with radiation therapy). Thirteen patients finished the post-operative treatment as scheduled, while three withdrew agreement to continue BRAF/MEK inhibitor therapy during the post-operative adjuvant period. Only in two cases was the adjuvant part of the therapy finished due to toxicity, while 13 were still on treatment at data cut-off. Progression of the disease during postoperative treatment was detected in fourteen cases. After treatment in the follow-up period, the most common recurrences were central nervous system metastases—detected in 8 patients. Among all 23 patients who experienced PD, 16 received a second line of treatment, most often immunotherapy with pembrolizumab (nine cases). 14 patients had died at the time of analysis.

## 4. Discussion

BRAFi/MEKi therapy is related to a high pathological response rate in borderline resectable stage III and IV *BRAF*-mutated melanoma patients. At the same time, preclinical and clinical data in melanoma and other malignancies suggested that the neoadjuvant treatment approach is effective, which justifies further analyses and trials [33]. In this analysis, we present the largest cohort of melanoma patients treated with a targeted neoadjuvant approach in routine clinical practice, outside of clinical trial. In conclusion, we underline that important information that needs to be considered in neoadjuvant treatment are the response rate, time to response needed before the surgery, and the efficacy of the neoadjuvant treatment, including recurrence-free survival (RFS) and subsequent adjuvant therapy planning [13].

First data on the neoadjuvant treatment efficacy came from case reports on borderline resectable metastatic melanoma. The first reported case was a melanoma patient with left axilla and neck tumors treated with vemurafenib who achieved 50% tumor volume response and was qualified for a modified radical neck and axillary dissection [34]. In the second reported case, a patient with axillary lymph node metastasis was described. This patient was also treated with vemurafenib, which enabled an axillary lymph node dissection [35]. Subsequently, more cases and finally single trials were reported before our current report. Before now, three trials have defined the background for further research in the neoadjuvant field. In the Combi-Neo (NCT02231775) trial, patients were randomized 2:1 to receive neoadjuvant BRAF/MEK inhibition for 12 weeks followed by resection and up to 44 weeks of post-operative treatment, for a total of 52 weeks, versus up-front surgery. The active treatment arm is concordant with our treatment strategy. After 18 months of follow-up, 71% (10/14) of patients in the treatment arm remained free of disease while none were in the surgery-only arm. The radiologic response rate was 85%, and pathological CR was 58% [28]. In a single-arm phase II Neo-Combi (NCT01972347), patients with resectable stage III melanoma also received 12 weeks of neoadjuvant therapy before surgical resection and 40 weeks of adjuvant dabrafenib and trametinib therapy after that. In this study, 35 patients were enrolled, 49% (17/35) achieved a pCR, and the next 51% (18/35) had a pathologic partial response (pPR). Median distant metastasis-free survival (DMFS) was 30.8 months in the overall population, including 38.0 months in patients with a pCR, but only 27.7 months in those with pPR. The 2-year OS was 93.8%, while median OS was not reached, but 57% (20/35) patients recurred, including eight cases with brain metastases [30]. Finally, in the REDUCTOR trial, eight-week treatment with dabrafenib and trametinib was used to study the conversion rate from unresectable to resectable tumors in patients with locally advanced stage III or oligometastatic stage IV melanoma. Among 21 recruited, two progressed and could do not undertake surgery, 16 had R0 resection, and one had a R1 resection. In the surgery group pCR was achieved in 35% (7/16), pPR in 35%, and no response in three, i.e., 15%. The 2-year OS was 84% [10]. As a consequence of the above-mentioned trials, in a recent pooled analysis from the International Neoadjuvant Melanoma Consortium covering 192 patients, only 51 received neoadjuvant targeted therapy. In this subgroup, complete pathological response (pCR) was achieved in 47% of cases. The presence of pCR correlated with improved RFS and OS. In the pooled analysis, a pCR was observed in 55% of patients treated with dabrafenib and trametinib. After a median follow-up of 10.2 months, 30% of patients had recurred. Only 18% of patients with pCR after neoadjuvant therapy have recurred, while 44% without pCR recurred. Nevertheless, in patients obtaining pCR on targeted therapy, the 2-year RFS was only 79%, and OS was only 91%, which was lower than data for neoadjuvant immunotherapy [36].

Although there are multiple possibilities for surgery and systemic therapy timing, there are several important advantages of neoadjuvant therapy. First of all, neoadjuvant treatment generates an opportunity to decrease the volume of the tumor, make a surgical resection feasible, and provide better cosmetic outcomes with tumor-free margins [13]. In patients with unresectable disease, neoadjuvant therapy may down-stage the disease to a resectable size. In patients with borderline resectable tumors, neoadjuvant treatment is expected to improve operability. These benefits of neoadjuvant BRAFi/MEKi include reducing tumor size/burden, improving surgical resectability with organ preservation, and increased locoregional disease control rate and finally, improvement of overall survival of these melanoma patients. In our report, we have shown over 12 weeks targeted neoadjuvant therapy of melanoma, the risk of losing regional control or progression is low. Our data also confirms that some patients with unresectable disease are converted to resectable and a complete clinical and pathological response to treatment can be obtained in approximately 25% of patients. Neoadjuvant treatment reduced the disease volume and therefore facilitated subsequent surgical resection in our patients.

Other advantages of neoadjuvant therapy are reduction of delay in therapy initiation, high treatment completion rates, radiological along with pathological assessment of treatment response, and collection of tumor specimens for genetic and translational research [37]. As we have shown in the case of our patients, neoadjuvant treatment response may be considered as a surrogate biomarker of disease recurrence. Implementation of neoadjuvant therapy enables access to melanoma tumor before and during therapy, which allows pathology and molecular studies and therefore may guide adjuvant therapy or new drug development trials. After neoadjuvant treatment, tumor response to therapy may be evaluated and comparison of tissue obtained by biopsy before treatment and resected tumor tissue may be compared [38]. The pathologic and molecular examination of a resected tumor may also provide biological data on the potential mechanism of response or resistance to treatment used. In fact neoadjuvant therapy treated tumor examination enables an early evaluation of the effectiveness of targeted therapy. Further research on tumor samples may also facilitate the development of novel biomarkers [39]. First of all valuable prognostic biomarker information comes from the pathologic response observed. In fact neoadjuvant approach enables evaluation of the benefits of targeted therapy in a short time. In the future, obtained prognostic and predictive information should aid informed decisions on further therapies. Besides pathological response, novel predictive biomarker analysis of responders and non-responders could further help to determine which cases will benefit long-term from the neoadjuvant therapy. Biomarker analyses that were conducted concordantly with the neoadjuvant trials have suggested an important role for T cell-mediated immune response, but further research is needed [37]. It is only defined that achievement of pCR correlates with melanoma PD-L1 expression, CD8+ T-cell infiltration, and a higher number of Ki67-positive melanoma cells at baseline [30]. Several issues remain in question, including the optimal duration of neoadjuvant BRAF inhibition prior to surgery, the role of adjuvant therapy, whether imaging response correlates with pathologic response, whether pathologic response correlates with RFS and OS, and if responses can inform post-operative treatment decisions [15].

In our practice report, longer follow-up will be needed to estimate final OS benefit after neoadjuvant therapy, as well as to analyze the impact of further lines of treatment on the survival of our patients. In general, our analysis, as well as neoadjuvant targeted therapy trials, used BRAFi/MEKi preoperatively and later postoperatively for up to a year, and therefore it is not possible to answer the question of whether neoadjuvant therapy alone is potentially sufficient treatment in melanoma. In a single routine clinical practice report in regionally advanced and oligometastatic melanoma, 23 patients were analyzed. These patients received no adjuvant treatment after neoadjuvant therapy and surgery. Ten (44%) achieved a pathologic complete response (pCR). In this study, no correlation between RECIST response and pathologic response was found. In survival analysis after a median of 43-month follow-up, only one patient who achieved a pCR and eight without a pCR recurred. These authors concluded that patients with a pCR had significantly improved RFS and OS in patients with residual tumors [15].

## 5. Conclusions

Melanoma patients on neoadjuvant therapy should be managed by a multidisciplinary team and the use of neoadjuvant therapy for resectable melanoma patients should be discussed with the patient and potential risks and benefits explained, especially in a patient population in which some patients might be cured by standard available therapies. Targeted preoperative therapy is a safe and effective therapy with good long-term results, since BRAFi/MEKi therapy leads to responses in majority of patients in a short period of time, so it poses a high chance for increasing radical resectability and when combined with post-operative therapy is a viable treatment option/alternative instead of an up-front surgical approach followed by post-operative therapy. Neoadjuvant therapies are expected to change the standard of care in resectable high-risk and/or palpable stage III melanoma. The use of neo-adjuvant therapy may enable radical resection of tumors in patients with initially unresectable locally advanced cases.

## Figures and Tables

**Table 1:** Baseline patients characteristics.

Age		Mean (Median)	SD	Range
		50 (55)	17.91	17–84
LDH		209 (180)	78.84	128–513
NLR		2.9 (2.4)		0.78–0.73
Gender		*N*	%	
	F	26	56.5	
	M	20	43.5	
ECOG	0	24	52.2	
	1	21	45.7	
	2	1	2.2	
Melanoma	Skin	36		
	Mucosal	2		
	UPM	8		
Primary tumor location	Head and neck	4		
	Upper and lower limb	18		
	Chest	3		
	Abdomen	2		
	Back	9		
	Genito-urinary	2		
	UPM	8		
Primary tumor	T1	2		
	T2	6		
	T3	10		
	T4	17		
	Tx	3		
	UPM	8		
Lymph nodes	N0	12		
	N1a	0		
	N1b	7		
	N1c	6		
	N2a	0		
	N2b	5		
	N2c	3		
	N3a	0		
	N3b	13		
	N3c	0		

UPM—unknown primary melanoma.

**Table 2:** Disease stage at neoadjuvant treatment initiation.

Disease Location	Number of Patients	% of Patients
Localized disease	8	17.4
Skin metastases	5	10.9
Extra-regional nodes metastases	5	10.9
Regional nodes metastases	14	30.4
Recurrence after LND/SNLB	11	23.9
Primary tumor recurrence	3	6.5

LND—lymphadenectomy; SNLB—sentinel node lymph node biopsy.

**Table 3:** Surgery performed after neoadjuvant treatment.

Resection	*N*	%
Skin metastases resection	7	15.2
Extra-regional LND	3	6.5
Regional LND	18	39.1
Recurrence after LND/SNLB resection	11	23.9
Primary tumor scar recurrence resection	7	15.2

**Table 4:** PFS and OS rate in groups with major (<10% melanoma cells) and minor pathological responses after neoadjuvant treatment.

PFS	DFS	OS
<10% melanoma cells	<10% melanoma cells	<10% melanoma cells
12m = 94.7% (95%CI 0.852–1.000)	12m = 94.1% (95%CI 0.84–1)	12m = 100% (95%CI 1.000–1)
24m = 71.6% (95%CI 0.533–0.962)	18m = 80.7% (95%CI 0.63–1)	24m = 94.7% (95%CI 0.852–1)
36m = 43.0% (95%CI 0.211–0.874)	24m = 44.8% (95%CI 0.25–0.8)	36m = 75.8% (95%CI 0.483–1)
>10% melanoma cells	>10% melanoma cells	>10% melanoma cells
12m = 76.9% (95%CI 0.6232–0.949)	12m = 51.8% (95%CI 0.35–0.76)	12m = 84.6% (95%CI 0.718–0.997)
24m = 41.3% (95%CI 0.2509–0.681)	24m = 33.6% (95%CI 0.18–0.63)	24m = 56.8% (95%CI 0.373–0.865)
36m = 20.7% (95%CI 0.0474–0.902)	36m = 33.6% (95%CI 0.18–0.63)	36m = 56.8% (95%CI 0.373–0.865)

## Data Availability

All data generated or analyzed during this study are available upon reasonable request upon DTA consent.
